# Longitudinal Changes in Functional Connectivity of the Caudate Is Associated With Recovery From Bell’s Palsy

**DOI:** 10.3389/fnagi.2019.00295

**Published:** 2019-11-07

**Authors:** Sheng Hu, Hongxing Kan, Junling Kan, Chuanfu Li, Aihong Yuan, ChunSheng Xu, Anqin Wang, Yi Wang, Xuan Bao, Tongping Shen, Hongli Wu

**Affiliations:** ^1^Department of Medical Information Engineering, Anhui University of Chinese Medicine, Hefei, China; ^2^The First Affiliated Hospital of Anhui University of Chinese Medicine, Hefei, China

**Keywords:** Bell’s palsy, caudate, longitudinal changes, functional connectivity, functional magnetic resonance imaging

## Abstract

Several studies have demonstrated through resting-state functional magnetic resonance imaging (fMRI) that functional connectivity changes are important in the recovery from Bell’s palsy (BP); however, these studies have only focused on the cortico-cortical connectivity. It is unclear how corticostriatal connectivity relates to the recovery process of patients with BP. In the present study, we evaluated the relationship between longitudinal changes of caudate-based functional connectivity and longitudinal changes of facial performance in patients with intractable BP. Twenty-one patients with intractable BP underwent resting-state fMRI as well as facial behavioral assessments prior to treatment (PT) and at the middle stage of treatment (MT); and 21 age- and sex-matched healthy controls (HC) were recruited and received the same protocol. The caudate was divided into dorsal and ventral sub-regions and separate functional connectivity was calculated. Compared with HC, patients with intractable BP at the PT stage showed decreased functional connectivity of both the dorsal and ventral caudate mainly distributed in the somatosensory network, including the bilateral precentral gyrus (MI), left postcentral gyrus, media frontal gyrus, and superior temporal gyrus (STG). Alternatively, patients in the MT stage showed decreased functional connectivity primarily distributed in the executive network and somatosensory network, including the bilateral cingulate cortex (CC), left anterior cingulate cortex (LACC), inferior prefrontal gyrus (IFG), MI, STG, and paracentral lobe. The longitudinal changes in functional connectivity of both the dorsal and ventral caudate were mainly observed in the executive network, including the right ACC, left CC, and IFG. Functional connectivity changes in the right ACC and left IFG were significantly correlated with changes in facial behavioral performance. These findings indicated that corticostriatal connectivity changes are associated with recovery from BP.

## Introduction

Bell’s palsy (BP) is a common disorder caused by an idiopathic peripheral facial paralysis; patients generally completely recover within 6 months without requiring medication (Engström et al., 2008). Several studies have demonstrated that functional connectivity changes are associated with the functional improvements of the facial nerve during the course of BP rehabilitation. A single-case study has demonstrated that in a patient with BP, recovery from facial nerve palsy was complemented by cortical reorganization (Klingner et al., 2012). Our previous study used resting-state functional connectivity to investigate the relationship between the function of the anterior cingulate cortex (ACC) and the course of BP. The functional connectivity strength of the ACC has been shown to enhance the prolonged concomitant course of disease (Hu et al., 2015). However, these studies have only focused on functional connectivity within the cerebral cortex and little is known about how changes of functional connectivity in subcortical regions, such as the basal ganglia nucleus, are related to patients’ recovery from BP.

The caudate is one of the structures that make up the dorsal striatum, which is involved in the integration of spatial information with motor behavior formulation and contributing to body posture, and the speed and accuracy of directed movements through both the corticostriatal loop and the dopamine system (Postle and D’Esposito, 1999, 2003). Previous neuroimaging studies of the dorsal striatum suggest that disrupted connectivity of the caudate can cause motor disorders, such as Parkinson’s disease and Huntington’s diseases (McColgan et al., 2015; Ji et al., 2018), which are influenced by the degeneration of the dopamine system (Stoessl et al., 2014). This demonstrated that the caudate controls regular movement by modulating the dopamine system and is important in the development of motor diseases. Since these studies analyzing the function of caudate were conducted in patients with brain damage, it is difficult to explore the functional recovery modulated by the caudate without an intact dopamine system.

BP presents an opportunity to investigate the function of the caudate in motor disorders with an intact dopamine system. The palsy is a purely unilateral deafferentation of facial muscles; it incites damages to the facial never that transfers signals from the motor cortex to the facial muscles (Klingner et al., 2014). While BP does not affect the trigeminal nerves that carry somatosensory afference, the brain can detect such a sensory-motor mismatch mistake. Compared to other diseases, such as Parkinson’s disease, BP does not impair brain structure and leaves the dopamine system unaffected (Vakharia and Vakharia, 2016), thereby possibly allowing the brain to stimulate the dopamine system to modulate this sensorimotor mismatch error. The caudate is a crucial brain region that is highly innervated by dopamine neurons (Min et al., 2016). Therefore, we hypothesized that in BP, changes in functional connectivity of the caudate might be the result of the central nervous response to sensorimotor mismatch error in patients with BP, and these changes may promote patients’ rehabilitation.

In the present study, we performed a longitudinal cohort study of intractable BP patients who had been treated for longer than 3 months and had not been in recovery prior to recruitment based on their caudate-based connectivity. First, we investigated how functional connectivity of the caudate is disrupted in patients with BP compared with healthy controls (HC). Second, we examined longitudinal functional connectivity changes of the caudate between patients prior to treatment (PT) and those in the middle stage of treatment (MT). Finally, we evaluated how the altered longitudinal functional connectivity of the caudate was associated with the improvements in facial function performance of patients with intractable BP.

## Materials and Methods

### Participants

Twenty-one patients with intractable BP (left side palsy: 8; mean age: 38.52 ± 3.66 years; 16 females) whose disease duration was longer than 3 months were recruited from the First Affiliated Hospital of Anhui University of Chinese Medicine (AUCM), and 21 age- and sex-matched HC (mean age: 37.89 ± 2.67 years; 15 females) were recruited from the local community. Patients who presented with neurological or psychiatric diseases, poorly controlled hypertension, or head injury were excluded. Additional exclusion criteria were pregnancy, substance abuse, or any other condition that might affect the study. All participants gave written informed consent prior to the study that was approved by the Ethics Committee of the First Affiliated Hospital of AUCM.

To exclude the influence of other factors, patients with BP were only permitted to have received acupunctural treatment. Patients received acupunctural treatment using the method of combinations of acupoints three times a week with 30 min each time. Patients in MT stage have been treated for at least 5 weeks.

### Clinical Assessment of Facial Function

We use the Sunnybrook grading system and House-Brackmann (H-B) grading systems to assess the severity of facial palsy in patients with intractable BP. The assessments of facial function are performed at rest and during movements of five facial expressions (lifting the eyebrows, closing eyes, wrinkling the nose, smiling and puckering of the lips). The score on the Sunnybrook ranges from 0 (representing complete paralysis) to 100 (represents normal facial function). The H-B grading system is divided into six grades to evaluate the facial function, where I represents normal facial function and VI represents complete paralysis. PT, patients with BP suffer from moderately severe facial dysfunction (H-B IV) and are often unable to lift the eyebrows, completely close the eyes, or keep mouth symmetry. Patients with BP at MT often demonstrate mild (H-B II) or moderate (H-B III) facial dysfunction.

In order to further understand the pathological stage of BP, we measured the electroneurography in the muscles that the facial nerve innervates. The neuroelectrophysiological apparatus (Japan Photoelectric MEB-9100k) was used to examine the neuromyogram of patients at PT and MT stages, respectively. The amplitudes and latency of motor evoked potentials of temporal, zygomatic, and mandibular branch of affected side nerve were recorded, respectively.

### Image Acquisition

Magnetic resonance imaging (MRI) scans were initially performed at the PT stage and at follow-up in the MT stage. MRI data were acquired using a 3.0-Tesla MR system (Discovery MR750, General Electric) with an eight-channel high-resolution radio-frequency head coil. Sagittal 3D T1-weighted images were acquired using a T1-3D BRAVO sequence with repetition time (TR)/echo time (TE): 8.2 ms/3.2 ms, flip angle (FA): 12°, matrix: 256 mm × 256 mm, field of view (FOV): 256 mm × 256 mm, slice thickness: 1 mm, with 166 axial slices with no gap. Resting-state functional MRI (fMRI) images were acquired using a gradient-echo single-shot echo planar imaging sequence with TR/TE: 2,000/35 ms, FOV: 240 mm × 240 mm, matrix: 64 × 64, FA: 90°, slice thickness: 3 mm, with 130 volumes. During the scanning, all participants were instructed to lie down with their eyes closed, to keep their mind blank, and not fall asleep.

### Data Preprocessing

For each participant, resting-state fMRI images were preprocessed by using a combination of analysis packages, including FSL^1^ and AFNI^2^. Before data preprocessing, all patients with left side palsy were flipped along the y-axis (R-L flip; Klingner et al., 2014). Functional images were preprocessed using the following steps: (1) the first 10 functional images were discarded to eliminate transients and account for T1 relaxation effects, followed by slice timing to compensate for acquisition delays across slices; (2) motion correction was performed by realigning all functional images to the middle image and the data with head motion over 2 mm or 2° were excluded; (3) functional images were co-registered to the high-resolution anatomical images, and then normalized to Montreal Neurological Institute standard brain; (4) voxels were re-sampled to 2 × 2 × 2 mm resolution; (5) images were spatially smoothed with a 6 mm full-width at half-maximum (FWHM) Gaussian kernel; (6) the data were linearly detrended, and the residual signals were band-pass temporal filtered at 0.01–0.1 Hz; and (7) nuisance variable regression was performed to regress out the six head motion parameters and the signals of white matter (WM) and cerebrospinal fluid (CSF).

### Statistical Analysis of Behavior

Statistical analyses of facial function (Sunnybrook scale) were performed using SPSS version 23 (Statistical Package for the Social Sciences; IBM, New York, NY, USA). Group level two-sample *t*-tests were performed to investigate whether patients in the MT stage would exhibit better performance relative to patients in the PT stage.

### Functional Connectivity Analysis

**Figure 1:**
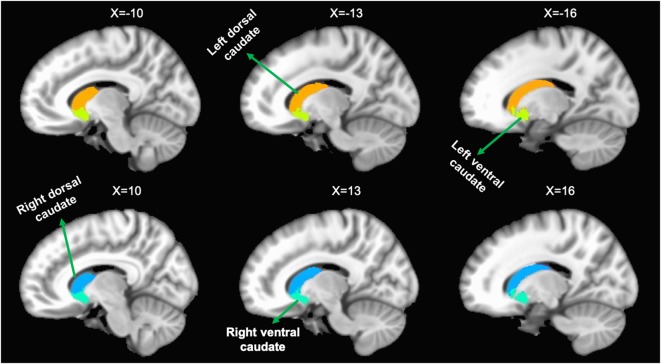
Representation of the four caudate regions of interest. The above panel shows the left dorsal caudate and left ventral caudate onto sagittal brain views. The bottom panel shows the right dorsal caudate and right ventral caudate onto sagittal brain views.

In order to evaluate longitudinal functional connectivity changes of the caudate, we selected the bilateral caudate as seeds, which were divided into dorsal and ventral sub-regions. The seeds were extracted from a probabilistic connectivity striatal atlas found in the FSL package^3^. The caudate seeds are shown in Figure 1.

For each participant, the Pearson correlation coefficient between the mean time series of bilateral caudate seeds and the time series of every voxel across the whole brain was calculated and converted to a *z*-value using Fisher r-to-z transformation to improve the normality.

### Statistical Analysis of Functional Connectivity

A group analysis was first carried out using a two-sample *t*-test to compare functional connectivity based on each caudate seed between patients with BP and HC. To evaluate longitudinal changes, we then performed a paired two-sample *t*-test to compare functional connectivity based on each caudate seed between patients at the MT and PT stages. The results of group analysis were corrected using a false discovery rate of *p* < 0.01.

### Correlation Analyses

We explored the correlation between longitudinal functional connectivity changes and facial function improvements in the patient group. For all correlation analyses, we used partial correlations to factor out age and sex, and *p* < 0.05 was considered to be statistically significant.

## Results

### Facial Function Measures

**Figure 2:**
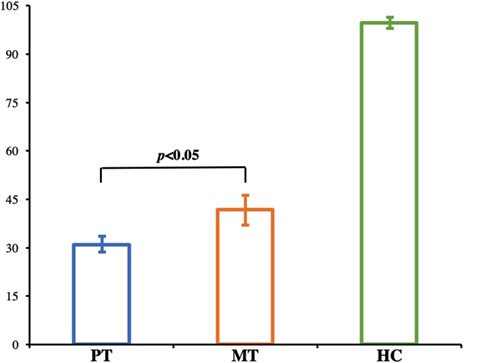
Bar graph shows the facial performance scale in HC and patients with BP. Normal facial functional performance of HC was presented more significantly than that of patients in the MT and PT stages, and patients in MT stage presented significantly better performance compared to patients in PT stage (*p* < 0.05). HC, Healthy Controls; PT, patients with BP prior to treatment; MT, patients with BP at middle stage of treatment.

A longitudinal analysis was conducted to assess changes in facial function as a function of recovery. Normal facial functional performance of HC was presented more significantly than that of patients in the MT and PT stages (*p* < 0.05, Figure 2). Patients in the MT stage presented significantly better performance on the Sunnybrook scale compared to patients in the PT stage (*p* < 0.05, Figure 2); however, no significant changes in electroneurography between patients with BP in the MT and PT stages were found (*p* > 0.05, Table 1).

**Table 1:** The comparison of electroneurography between patients with BP in the MT and PT stages.

Facial nerve	Stage	AMEPs (mv)	LMEPs (ms)
Zygomatic branch	PT	1.50 ± 0.58	3.28 ± 1.22
	MT	1.75 ± 0.59	2.7 ± 0.36
Mandibular branch	PT	1.94 ± 0.88	2.55 ± 0.41
	MT	1.94 ± 0.64	2.73 ± 0.44
Temporal branch	PT	1.36 ± 0.76	3.02 ± 0.48
	MT	1.31 ± 0.45	2.88 ± 0.55

*Abbreviation: AMEPs, Motor evoked potentials amplitude; LMEPs: Motor evoked potentials latency*.

### Group Comparison of Functional Connectivity Between Patients in the PT Stage and HC

**Figure 3:**
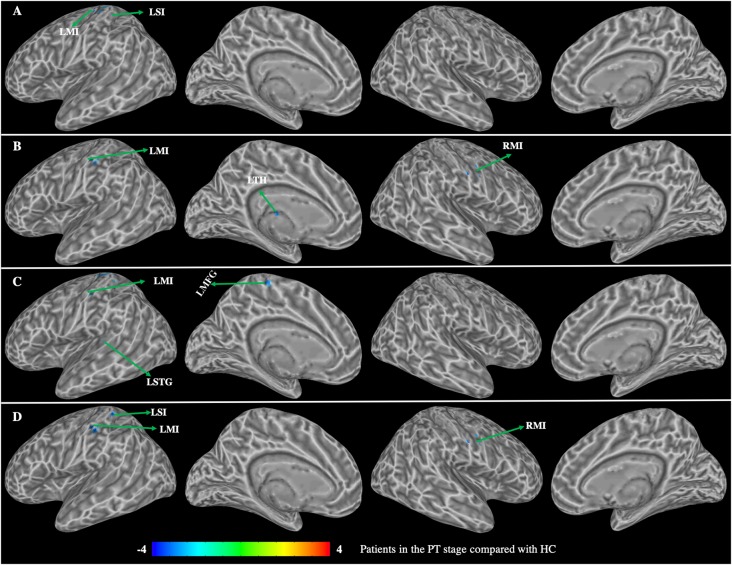
Group differences in functional connectivity between patients with BP in the PT stage compared with HC (*p* < 0.01, corrected with false discovery rate). **(A)** Functional connectivity of the left dorsal caudate; **(B)** functional connectivity of the left ventral caudate; **(C)** functional connectivity of the right dorsal caudate; **(D)** functional connectivity of the right ventral caudate. LMI, left precentral gyrus; LSI, left postcentral gyrus; LSTG, left superior temporal gyrus; LTH, left thalamus; LMFG, left medial frontal gyrus; RMI, right prefrontal gyrus.

Patients in the PT stage showed decreased functional connectivity of the caudate, which were mainly distributed in the somatosensory motor network (Figure 3, Table 2). Specifically, compared with HC, patients in the PT stage showed significantly decreased functional connectivity of both the left and right dorsal caudate to regions of the left precentral gyrus (MI). Patients in the PT stage also showed significantly decreased functional connectivity of both the left and right ventral caudate to regions of bilateral MI. In addition, we found multiple regions of functional connectivity that were spatially distinct between the left and right caudate maps. Specifically, we additionally found decreased functional connectivity of the left dorsal caudate to the left postcentral gyrus (SI), while decreased functional connectivity of right dorsal caudate was additionally found in the left medial frontal gyrus (MFG) and superior temporal gyrus (STG). The left ventral caudate showed differences in the decreased functional connectivity to left thalamus, and the right ventral caudate demonstrated decreased functional connectivity to the left SI.

**Table 2:** Group differences in functional connectivity between patients with BP in the PT stage compared with healthy controls (HC).

Seed	Regions	Side	MNI Coordinate			Voxel	*z*-value
			*x*	*y*	*z*		
Left dorsal caudate	MI	L	−26	−24	68	53	−4.32
	SI	L	−38	−32	56	52	−3.55
Left ventral caudate	MI	L	−46	−20	44	55	−4.15
	MI	R	58	−2	32	30	−4.24
	Thalamus	L	−6	−16	14	23	−3.72
Right dorsal caudate	MI	L	−28	−34	58	112	−3.89
	MFG	L	−6	−28	58	27	3.66
	STG	L	−48	−30	6	20	−3.42
Right ventral caudate	MI	L	−46	−20	42	82	−4.12
	SI	L	−36	−40	58	61	−3.73
	MI	R	58	−2	32	26	−3.76

*Abbreviation: MI, precentral gyrus; SI, postcentral gyrus; STG, superior temporal gyrus; TH, thalamus; MFG, medial frontal gyrus; L, left; R, left*.

### Group Comparison of Functional Connectivity Between Patients in the MT Stage and HC

**Figure 4:**
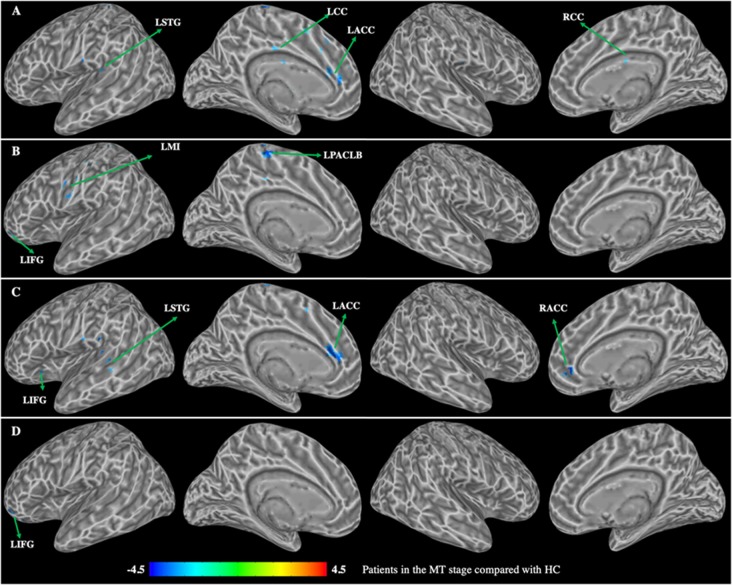
Group differences in functional connectivity between patients with BP in the MT stage compared with HC (*p* < 0.01, corrected with false discovery rate). **(A)** Functional connectivity of the left dorsal caudate; **(B)** functional connectivity of the left ventral caudate; **(C)** functional connectivity of the right dorsal caudate; **(D)** functional connectivity of the right ventral caudate. LSTG, left superior temporal gyrus; LMI, left precentral gyrus; LIFG, left inferior frontal gyrus; LCC, left cingulate cortex; RCC, right cingulate cortex; LACC, left anterior cingulate cortex; RACC, right anterior cingulate cortex; LPACLB, left paracentral lobe.

Patients in the MT stage showed decreased functional connectivity of the caudate, which was mainly distributed in the executive and somatosensory network (Figure 4, Table 3). Specifically, compared with HC, patients in the MT stage demonstrated significantly decreased functional connectivity of both the left and right dorsal caudate to regions of left ACC and STG. Additionally, we found decreased functional connectivity of the left dorsal caudate to the bilateral cingulate cortex (CC), and found decreased functional connectivity of the right dorsal caudate to regions of the right ACC and left inferior frontal gyrus (IFG). Patients in the MT stage demonstrated significantly decreased functional connectivity of both the left and right ventral caudate to the left IFG. In addition, decreased functional connectivity between the left ventral caudate and regions, including the left paracentral lobe (PACLB) and left MI was also observed in patients at the MT stage.

**Table 3:** Group differences in functional connectivity between patients with BP in the MT stage compared with HC.

Seed	Regions	Side	MNI Coordinate			Voxel	*z*-value
			*x*	*y*	*z*		
Left dorsal caudate	ACC	L	−4	44	12	110	−3.91
	STG	L	−46	−36	8	28	−3.13
	CC	L	−2	−10	28	23	−3.11
	CC	R	0	−2	46	43	−3.5
Left ventral caudate	PACLB	L	−6	−26	58	79	−4.44
	IFG	L	−34	40	−14	54	−3.49
	MI	L	−60	−4	32	42	−3.71
Right dorsal caudate	ACC	L	−4	−42	12	182	−4.12
	STG	L	−50	−28	16	50	−3.58
	IFG	L	−28	28	−6	21	−3.51
	ACC	R	10	38	−4	20	−4.0
Right ventral caudate	IFG	L	−42	34	−10	86	−4.3

*Abbreviation: STG, superior temporal gyrus; MI, precentral gyrus; IFG, inferior frontal gyrus; CC, cingulate cortex; ACC, anterior cingulate cortex; PACLB, paracentral lobe. L, left; R, right*.

### Longitudinal Functional Connectivity Changes in Patients With BP

**Figure 5:**
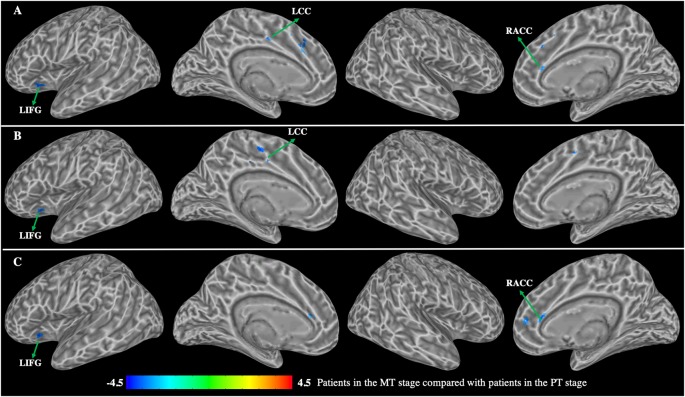
Group differences in functional connectivity between patients with BP in the MT stage compared with patients with BP in the PT stage (*p* < 0.01, corrected with false discovery rate).** (A)** Functional connectivity of the left dorsal caudate; **(B)** functional connectivity of the left ventral caudate; **(C)** functional connectivity of the right dorsal caudate. LIFG, left inferior frontal gyrus; LCC, left cingulate cortex; RACC, right anterior cingulate cortex.

Patients in the MT stage exhibited significantly decreased functional connectivity of the caudate primarily observed in the executive network relative to patients in the PT stage (Figure 5, Table 4). Specifically, patients in MT stage illustrated significantly decreased functional connectivity of both the left and right dorsal caudate to regions of left IFG and right ACC relative to patients in the PT stage. Additionally, decreased functional connectivity of the left dorsal caudate to the left CC was also found in patients at the MT stage relative to patients at the PT stage. Patients in the MT stage showed decreased functional connectivity of the left ventral caudate to regions of the left IFG and CC; however, no significant functional connectivity changes of the right ventral caudate were demonstrated.

**Table 4:** Group differences in functional connectivity between patients with BP in the MT stage compared with patients with BP in the PT stage.

Seed	Regions	Side	MNI Coordinate			Voxel	*z*-value
			*x*	*y*	*z*		
Left dorsal caudate	IFG	L	−32	24	−10	56	−3.42
	CC	L	−2	−2	44	28	−3.7
	ACC	R	4	34	16	24	−3.01
Left ventral caudate	IFG	L	−28	22	−12	59	−3.19
	CC	L	−4	−16	46	67	−3.75
Right dorsal caudate	ACC	R	4	36	16	82	−3.8
	IFG	L	−30	24	−10	52	−3.76

*Abbreviation: IFG, inferior frontal gyrus; CC, cingulate cortex; ACC, anterior cingulate cortex; L, left; R, right*.

**Figure 6:**
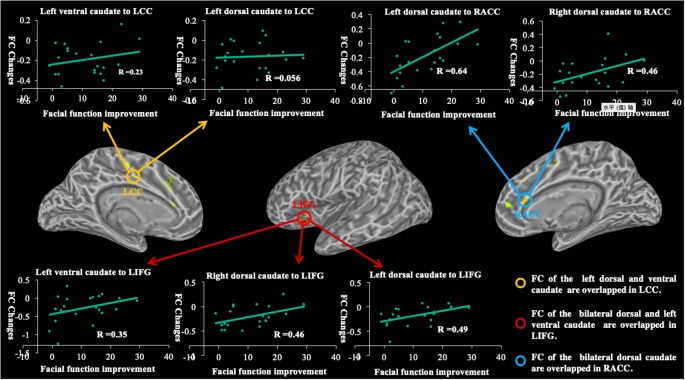
The graph shows the overlapped brain regions in the longitudinal changes of caudate-based functional connectivity and demonstrates relationships between functional connectivity changes and facial function improvements in patients with BP. R, coefficient of correlation; FC, functional connectivity; LIFG, left inferior frontal gyrus; LCC, left cingulate cortex; RACC, right anterior cingulate cortex.

The decreased functional connectivity overlap among caudate seeds is shown in Figure 6. Functional connectivity decreases in the bilateral dorsal and left ventral caudate are largely overlapped (red) in the left IFG. The decreased functional connectivity in the right ACC overlaps (yellow) with bilateral dorsal caudate-based functional connectivity maps. The overlap (yellow) in decreased functional connectivity of the left dorsal and ventral caudate is shown in the left CC.

### Correlation Between Longitudinal Changes of Functional Connectivity and Facial Function Improvements

The correlations between longitudinal functional connectivity changes of each caudate seed and facial function improvement are shown in Figure 6. We found significant correlations between longitudinal functional connectivity changes of the left dorsal caudate (*r* = 0.64, *p* = 0.0018) and right (*r* = 0.46, *p* = 0.0367) dorsal caudate to the right ACC and facial function improvement. The significant correlations between longitudinal functional connectivity changes of the left dorsal caudate (*r* = 0.49, *p* = 0.0256), right dorsal caudate (*r* = 0.46, *p* = 0.0356) and left ventral caudate (*r* = 0.35, *p* = 0.1222) to the left IFG and facial function improvement were also found. However, no significant correlations between functional connectivity of the left dorsal caudate (*r* = 0.056, *p* = 0.808) and left ventral caudate (*r* = 0.23, *p* = 0.3055) to the left CC and facial function improvement were found in the current study.

## Discussion

To the authors’ knowledge, the present study is the first to investigate the longitudinal changes in the functional connectivity of the caudate in patients with intractable BP. Results suggested that longitudinal functional connectivity changes of the caudate may be able to serve as a neuroimaging biomarker in the recovery from BP. The longitudinal functional connectivity changes of the caudate were mainly observed in the right ACC, left CC and IFG. Additionally, we found that the functional connectivity changes in right ACC and left IFG were significantly associated with the recovery from facial palsy.

### Caudate-Based Functional Connectivity Changes of BP Comparison With HC at PT Stage

Neuroimaging studies suggest that the caudate integrates spatial information with motor behavior formulation (Postle and D’Esposito, 1999, 2003). The disrupted functional connectivity between the caudate and somatosensory motor regions can cause motor deficit disorders, such as Parkinson’s and Huntington’s diseases (Douaud et al., 2006; Hacker et al., 2012; Manza et al., 2016; Yang et al., 2016). Our results demonstrate that patients with BP in the PT stage show significantly decreased functional connectivity between the caudate and somatosensory motor regions compared with HC. Previous findings illustrated that corticostriatal connections are significantly enhanced in patients with BP than HC at the acute stage (compared to HC) and demonstrated that the connections may be enhanced by the compensatory mechanisms for the sensorimotor mismatch (Song et al., 2017). The reason for the inconsistency may be that the corticostriatal dopamine path in patients with intractable BP is deficient compared to patients at the acute stage, therefore, resulting in brain state changes to adapt to the current disease state. Another previous study has reported that patients with BP were in different brain states as the disease progressed (Li et al., 2013). This indirectly indicates that the decreased functional connectivity among the somatosensory motor network was dependent on the current brain state regulated by the deficient dopamine system.

### Caudate-Based Functional Connectivity Changes of BP at the MT Stage Compared With HC

By systematically investigating the functional connectivity of the striatum, the dorsal caudate is primarily connected with the dorsolateral prefrontal cortex and ventrolateral prefrontal cortices, ACC, and posterior cingulate, whereas the ventral caudate is functionally correlated with the dorsolateral prefrontal cortex, IFG and posterior cingulate cortex (Leh et al., 2007; Draganski et al., 2008; Robinson et al., 2012). Additional, neuroimaging studies have proven that the caudate is connected with the prefrontal cortex and ACC is involved in the speed and accuracy of direct movement (Robinson et al., 2012). In the present study, we found that significantly decreased functional connectivity of the dorsal caudate was mainly distributed in the executive network in patients with BP at the MT stage, including the bilateral ACC, CC and left IFG, whereas decreased functional connectivity of the ventral caudate was mainly distributed in the left IFG. A potential mechanism for the changes is that a brain communication transfer occurs in patients with BP (Stam, 2014); in the recovery stage of BP, this likely occurs particularly in hub-regions to relieve the hub overload and begin to reroute information path to the node (Xu et al., 2018). The mechanism could explain how the caudate connection of BP changes from somatosensory motor network in the PT stage to executive network in the MT stage. Meanwhile, patients with BP in the MT stage showed significant improvements in facial expressions compared to patients in the PT stage. These findings imply that decreased functional connectivity in these brain regions might be related to patients’ recovery from BP.

### Longitudinal Changes in Caudate-Based Functional Connectivity

Neuroimaging studies have reported that longitudinally enhanced functional connectivity has been found in patients with BP (Klingner et al., 2012; Li et al., 2013; He et al., 2014). In a recent longitudinal study of patients with BP, investigators showed that functional connectivity of the cerebellar increased following the recovery course of facial motor functions (Smit et al., 2010). In our previous study, we found resting state functional connectivity increased between the ACC and other brain regions such as primary motor cortex, supplementary motor area, secondary somatosensory cortex, and middle cingulate cortex with increased duration of BP (Hu et al., 2015). These studies illustrated that functional connectivity changes occurred in BP brain, which play an important role in recovery from BP. Interestingly, we observed longitudinal functional connectivity decreases in patients with BP, which was primarily distributed in the executive network. Specifically, patients in the MT stage showed significant decreased functional connectivity of the dorsal caudate compared to patients in the PT stage in the left CC, IFG, and right ACC, and showed decreased functional connectivity of the ventral caudate in left IFG and CC. The decreased functional connectivity in the ACC, CC, and IFG was not observed in patients with BP at the PT stage compared to HC, while it was observed in patients at the MT stage. These findings indicate that corticostriatal connectivity decreases in the executive network might be a potential mechanism for improved facial function during recovery from BP.

### Relationship Between Longitudinal Changes of Caudate-Based Functional Connectivity and Facial Function Improvement

The relationship between the pattern functional connectivity and facial performance in patients with BP has been reported (Smit et al., 2010; Bian et al., 2016). Patients in the MT stage showed better facial function performance than patients in the PT stage; however, there were no significant changes in electroneurography between the two pathological stages of BP in the present study. Several studies have reported that using electromyography to identify severe cases of BP must be done in a timely manner because decompression of the facial nerve later than 14 days after onset of symptoms does not alter the prognosis of BP (Gantz et al., 1999; Zandian et al., 2014). These indicate that patients exhibiting greater axonal degeneration may be due to the transportation of the electromyographic signal. Additionally, patients with intractable BP had a disease duration of longer than 3 months. Therefore, it is possible that the structure of the facial nerve in patients with intractable BP is impaired, which results in significant changes in facial performance, but no changes in electroneurography between the two pathological stages of BP.

The longitudinal functional connectivity changes of the caudate were primarily found in the executive network, including the ACC, CC, and IFG, which may be related to patients’ recovery from BP. Specifically, we found significant linear correlations between functional connectivity changes in the ACC, IFG and changes in facial behavioral performance; however, no significant linear correlations were seen between functional connectivity changes in the CC and changes in facial behavioral performance. Previous studies have demonstrated that the dorsal caudate showed functional connectivity with the ACC and IFG involved in motor control and perception-related processes (Robinson et al., 2012). Therefore, we suppose significant correlations between functional connectivity changes in the executive network and changes in facial behavioral performance might be the consequence of the recovery process in patients with BP. These findings indicated that longitudinal functional connectivity changes in the executive network are associated with the recovery process in patients with BP, and could serve as a neuroimaging biomarker following patients’ recovery from BP.

## Conclusions and Limitations

The present study investigated the longitudinal functional connectivity changes of the caudate in patients with intractable BP. The data demonstrated that the longitudinal functional connectivity changes were primarily distributed in the executive network, which were significantly correlated with facial function improvements in patients with intractable BP. These findings indicated that decreased corticostriatal connectivity in the executive network could serve as a neuroimaging biomarker for the prognosis of facial function following recovery from BP.

There are some limitations to this study. First, we measured the electroneurography in the muscles with facial nerve innervates; however, there were no changes between the two pathological stages of BP, and it is unclear how the electroneurography influenced the brain network. Going forward, we should add a patients group who are in acute stage to study why there are no changes in electroneurography in patients with intractable BP and the relationship between electroneurography and neural networks. Second, it is difficult to prove the corticostriatal reorganization correlated with recovery from BP. Therefore, we need to further assess preclinical studies to better understand the mechanisms behind these alterations in future studies.

## Data Availability Statement

The raw data supporting the conclusions of this manuscript will be made available by the authors, without undue reservation, to any qualified researcher.

## Ethics Statement

The studies involving human participants were reviewed and approved by The Human Research Committee of the First Affiliated Hospital of Anhui University of Chinese Medicine. The patients/participants provided their written informed consent to participate in this study.

## Author Contributions

SH performed the experiment, analyzed image data, and drafted the manuscripts. HK and JK analyzed image data and gave critical comments on the manuscript. CL, AY, CX, and AW performed the experiment and collected the data. HW designed the study and gave critical comments on the manuscript. YW, XB, and TS helped to revise the manuscript.

## Conflict of Interest

The authors declare that the research was conducted in the absence of any commercial or financial relationships that could be construed as a potential conflict of interest.

## Footnotes

^1^https://fsl.fmrib.ox.ac.uk

^2^https://afni.nimh.nih.gov/download

^3^https://fsl.fmrib.ox.ac.uk/fsl/fslwiki/Atlases/striatumconn
